# Immune System-Related Plasma Pathogenic Extracellular Vesicle Subpopulations Predict Osteoarthritis Progression

**DOI:** 10.3390/ijms252312504

**Published:** 2024-11-21

**Authors:** Xin Zhang, Sisi Ma, Syeda Iffat Naz, Erik J. Soderblom, Vaibhav Jain, Constantin Aliferis, Virginia Byers Kraus

**Affiliations:** 1 Duke Molecular Physiology Institute, Duke University School of Medicine, Durham, NC 27701, USA; vaibhav.jain@duke.edu (V.J.); kraus004@duke.edu (V.B.K.); 2 Department of Orthopaedic Surgery, Duke University School of Medicine, Durham, NC 27701, USA; 3 Institute for Health Informatics, University of Minnesota, Minneapolis, MN 55455, USA; sisima@umn.edu (S.M.); iffat002@umn.edu (S.I.N.); califeri@umn.edu (C.A.); 4 Duke Proteomics and Metabolomics Core Facility, Duke University School of Medicine, Durham, NC 27701, USA; erik.soderblom@duke.edu; 5 Department of Medicine, Duke University School of Medicine, Durham, NC 27701, USA

**Keywords:** plasma, proteomics, flow cytometry, surface marker, predictor, progression

## Abstract

Certain molecules found on the surface or within the cargo of extracellular vesicles (EVs) are linked to osteoarthritis (OA) severity and progression. We aimed to identify plasma pathogenic EV subpopulations that can predict knee radiographic OA (rOA) progression. We analyzed the mass spectrometry-based proteomic data of plasma EVs and synovial fluid (SF) EVs from knee OA patients (n = 16, 50% female). The identified surface markers of interest were further evaluated in plasma EVs from an independent cohort of knee OA patients (n = 30, 47% female) using flow cytometry. A total of 199 peptides with significant correlation between plasma and SF EVs were identified. Of these, 41.7% were linked to immune system processes, 15.5% to inflammatory responses, and 16.7% to the complement system. Crucially, five previously identified knee rOA severity-indicating surface markers—FGA, FGB, FGG, TLN1, and AMBP—were confirmed on plasma EV subpopulations in an independent cohort. These markers’ baseline frequencies on large plasma EVs predicted rOA progression with an AUC of 0.655–0.711. Notably, TLN1 was expressed in OA joint tissue, whereas FGA, FGB, FGG, and AMBP were predominantly liver derived. These surface markers define specific pathogenic EV subpopulations, offering potential OA prognostic biomarkers and novel therapeutic targets for disease modification.

## 1. Introduction

The aging and senescence of the immune system contributes to systemic aging, the development of many age-related diseases, and increased morbidity and mortality [1,2]. A senescent immune system is reflected in extracellular vesicles (EVs); specifically, various molecules present on the surface or within the cargo, including DNAs, messenger RNAs, small RNAs, cytokines, mitochondria, and other proteomic components, as well as the frequencies of EVs carrying these molecules, reflect the pathophysiology of aging [3,4,5,6,7,8]. The cells that release EVs are referred to as their “parent cells”; a key feature of EVs is that they carry surface markers and cargo from their parent cells, enabling them to mediate intercellular communication with recipient cells; this occurs either through the transport of their cargo or via interactions between surface receptors and ligands [9,10,11,12,13]. Great advantages of EVs as biomarkers includes their stability in frozen biospecimens, ease of access, and ability to reflect parent immune cell populations allowing profiling in a “liquid biopsy” without the necessity of a fresh biospecimen of live cells. 

Osteoarthritis (OA) affects over 500 million people worldwide, with knee OA being the most prevalent form [14]. The prevalence of OA increases dramatically with age [15,16,17]. Many inflammatory factors were linked to the complicated pathological remodeling of connective tissues within the affected joints, leading to OA development and progression [18,19]. Our group and others have reported on the involvement of synovial fluid (SF) EVs in the pathogenesis of OA [20,21,22]. These findings highlight the potentiality of pathogenic SF EVs as a new generation of biomarkers for diagnostics and as targets for therapeutics in OA. However, the collection of SF is highly restricted in clinical practice due to the risks of bleeding, sterile inflammation, and infection associated with needle insertion into the joint space. Therefore, it is highly advantageous to identify systemic EV biomarkers that indicate OA severity and progression in a “minimally invasive” biospecimen (no biopsy required), such as plasma. It is of utility to identify biomarkers on the surface of plasma EVs that could be readily detected, for instance by flow cytometric methods, and targeted for quantification and modification for therapeutic purposes. 

Since all human cells can produce EVs, those in biofluids—especially in blood circulation—are highly heterogeneous, encompassing a wide variety of types, sizes, surface markers, and cargo [4,6]. While most studies in the EV research field have focused on small EVs (SEVs), such as exosomes, our group and others have aimed to study EVs ranging from small to large. This approach is important because the involvement of medium-sized EVs (MEVs) and large EVs (LEVs) in various processes, including the pathogenesis of OA, is under-evaluated. Previous studies have shown that LEVs, ranging from 1 to 10 μm, contain proteins and small RNAs, highlighting their potential for development as biomarkers in cancer [23]. LEVs produced by a natural killer cell line in vitro regulated the surface marker expression and function of monocyte/macrophage cell lines [24]. Moreover, some LEVs contain, transport, and release SEVs [25]. LEVs and SEVs share some common peptides and carry some peptides with differential abundance [26,27]. However, precisely isolating and effectively recovering LEVs and SEVs remain technically challenging. Based on size measurements using cryo-electron microscopy images, the diameters of LEVs and SEVs from the supernatants of in vitro cultures of four cell lines, separated by ultracentrifugation and density gradient, largely overlapped [26]. Similar size overlap was observed in another study that used various differential centrifugation forces (10,000× *g*, 18,000× *g*, and 100,000× *g*) to isolate EVs of different sizes [27]. Flow cytometry can gate LEVs and SEVs without physical isolation, allowing for the simultaneous measurement of their relative size, granularity distribution, and biomarker-specific subpopulations using fluorescence-conjugated antibodies or dyes. This technique provides an effective approach to analyze EVs of varying sizes in parallel [28,29]. Consistent with our previous report [20], a recent study isolated human regulatory macrophage-derived LEVs with a mean size of 7470 nm and detected typical EV surface markers (CD9, CD63, and CD81) on these LEVs using flow cytometry [30]. These results further support LEVs as a valuable subpopulation for study.

Using targeted analyses, we observed a positive correlation in the TNF-α concentration between plasma EVs and SF EVs from patients with knee OA [20]. Moreover, the frequencies of TNF-α^+^ LEVs, MEVs, and SEVs in plasma were predictors of the progression of radiographic osteoarthritis (rOA) [28], suggesting that plasma EVs could reflect joint-specific pathology. Our aim was to identify pathogenic EV subpopulations in “minimally invasive” biofluid plasma using EV surface markers. These surface markers indicate the parent cells of the EVs and provide accessible targets for isolating EV subpopulations from cells of interest, such as stem or progenitor cells, for use as autologous biological products. Additionally, they can facilitate the clearance of pathogenic EV subpopulations for OA therapy [4,31,32,33]. Based on these findings, we hypothesized the presence of plasma EV subpopulations with a positive correlation with matched OA SF EVs and specific surface markers that reflect the pathological conditions and disease activity of OA joints (i.e., progression risk). To test this hypothesis, we selected potentially pathogenic systemic EV candidates in OA plasma based on the following criteria: (1) a significant positive association with SF EV peptides, termed peptides^+(PL,SF)^; (2) predicted localization to the cell membrane; (3) association with OA disease severity based on our previous report [33]; (4) flow cytometric confirmed surface localization on plasma EVs in a separate cohort of knee OA patients; and (5) the ability to predict knee rOA progression.

Currently, no single method for isolating EVs offers both high-recovery and high-specificity while being suitable for all downstream analyses. All EV isolation methods, to varying degrees, capture subsets of EVs and non-EV particles. As a result, choosing an EV isolation technique that aligns with both the sample’s characteristics and the specific requirement of the downstream analysis is essential [34]. Ultracentrifugation is frequently used to isolate EVs, particularly SEVs like exosomes. However, it is more suitable for large-volume samples with simple or well-defined matrices, such as cell culture supernatants, and has drawbacks, including low EV yield and co-isolation of non-EV contaminants, especially when applied to biofluids [34,35,36,37,38]. For our studies using limited volumes of precious human biofluids, particularly from aged patient cohorts, we considered it essential to utilize an isolation method that guarantees a high yield of EVs. We chose polymer-based precipitation to maximize the yield and recovery of EVs from limited amounts of human specimens while preserving EV integrity and heterogeneity [20,28,33,34,36]. This method precipitates not only SEVs (exosomes), as reported by the commercial vendor, but also MEVs and LEVs, as observed in our previous studies [4,6,8,33] and others [39]. We consider this feature advantageous as it allows us to capture EVs across a broad range of sizes, facilitating a comprehensive characterization of EV particles and their biological correlates. To address concerns regarding the potential co-isolation of non-EV particles using this method, we centrifuged the biospecimens to eliminate unwanted material and debris before EV isolation and extensively characterized the isolated EVs using multiple established EV characterization techniques, as detailed in our previous publications, including nanoparticle tracking analysis, dynamic light scattering, transmission electron microscopy, and high-resolution multicolor flow cytometry [4,6,28]. For validation, we focused on EV-associated biomarkers that are not present in the most common non-EV contaminants and validated our findings using various methods and study cohorts. We confirmed that EVs isolated using this method have a lipid bilayer structure, a low frequency of the APOA1^+^ marker, and contain mitochondria and microRNAs [4,6,40]. EVs isolated from the same biospecimens as this study have previously been validated to exhibit a wide size distribution from small to large and carry EV and cell/tissue-specific surface markers (CD9, CD63, CD81, CD4, CD8, CD19, CD15, CD14, CD68, CD56, CD29, HLA-A/B/C, HLA-DR/DP/DQ, HLA-G, CD34, CD31, CD41a, and CD235a) [20,28]. OA SF EVs also carried OA joint cell-related surface markers, including CSPG4, CD109, VSIG4, CD163, MARCO, NRP1, LRP1, BGN, and PTPRS [33]. The frequencies of EV subpopulations carrying these markers vary dramatically [20,28,33], supporting the high heterogeneity of EVs in OA biofluid. 

## 2. Results

### 2.1. EV Characterization and Protein Cargo

EVs isolated from the same biospecimens as this study have previously been validated to exhibit a wide size distribution from small to large (see Methods Section 4 and Figure S1). Our recent proteomic analysis of EVs revealed 8396 peptides in plasma EVs and 8529 peptides in SF EVs from knee OA patients [33]. In the new analysis performed in the present study, we identified 203 peptides in plasma EVs that showed a significant correlation with the matched peptides in SF EVs: 98% (n = 199) of these peptides were positively correlated (termed herein peptides^+(PL,SF)^), whereas only 2% (n = 4) of these peptides were negatively correlated (termed herein peptides^+(PL,SF)^) (Table S1). Herein we mainly focused on EV peptides^+(PL,SF)^, for which higher plasma levels have the clearest potential to directly reflect joint pathogenic events in OA. STRING network analysis [41] indicated that proteins corresponding to EV peptides^+(PL,SF)^ are highly interactive (enrichment *p* < 1.0 × 10^−16^); among these identified proteins, 41.7%, 15.5%, and 16.7% were involved in immune system process, inflammatory response, and complement system, respectively; all functional pathways with established roles in OA pathogenesis. 

### 2.2. Surface EV Markers Correlated in OA Plasma and Synovial Fluid (Peptides^+(PL,SF)^)

We aimed to identify biomarkers on the surface of plasma EVs that could be easily detected by flow cytometric methods and potentially targeted for therapeutic purposes. For this goal, we focused on the newly identified 199 EV peptides^+(PL,SF)^, among which we identified 29 EV peptides^+(PL,SF)^ corresponding to 16 proteins with predicted surface location according to their Surface Protein Consensus (SPC) score ≥1 (reflecting surface localization indicated in one or multiple predictive datasets with higher SPC score indicating higher probability of surface localization [33,42]). Among these surface markers, seven EV peptides^+(PL,SF)^ showed a significant positive correlation with knee rOA severity scores; these included fibrinogen alpha chain (FGA), beta chain (FGB) and gamma chain (FGG) in SF, and protein AMBP (AMBP) and Talin-1 (TLN1) in plasma as we recently reported [33] (Table 1). 

**Figure 1:**
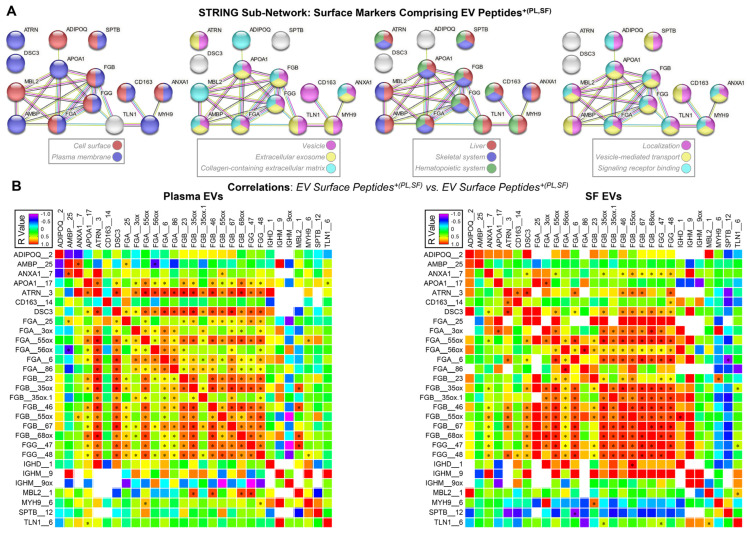
The EV surface peptides^+(PL,SF)^ from FGA, FGB, and FGG were highly correlated with each other in both plasma EVs and SF EVs. (**A**) The STRING networks display functional enrichments of 14 surface markers comprising the identified EV peptides^+(PL,SF)^ in membrane localization, cellular distribution, and tissue expression and function; the processes represented by the colors of each node in the network are detailed in the legend. Immunoglobulin components, IGHD and IGHM, are not mapped in the STRING network. The edges illustrate protein associations, both functional and physical, all supported by evidence. The reported pathways exhibited an enrichment False Discovery Rate (FDR, q value) < 0.05. (**B**) The associations of the EV surface peptides^+(PL,SF)^ with each other in plasma EVs and SF EVs were analyzed using Spearman correlations. Heat maps illustrate the correlation coefficients (r values); * indicated a significant result (*p* < 0.05).

Among the 16 predicted surface markers comprising 29 EV peptides^+(PL,SF)^, STRING network analysis [41] supported the localization of 14 markers on the cell surface and plasma membrane with the following characteristics: they were mainly expressed by the liver, skeletal system, and hematopoietic system; highly enriched in vesicles, extracellular exosomes, and collagen-containing extracellular matrix; and involved in localization, vesicle-mediated transport, and signaling receptor binding (Figure 1A). Notably, EV surface peptides^+(PL,SF)^ from FGA, FGB, and FGG were highly correlated with each other in both plasma EVs and SF EVs (Figure 1B), suggesting EVs carrying these surface markers may be released by the same type(s) of cells, predominantly hepatocytes, based on the Human Protein Atlas [43] and Tabula Sapiens [44,45]. 

### 2.3. FN1 Directly Interacts with Proteins Corresponding to 51.2% of Peptides Correlated in OA Plasma and Synovial Fluid (EV Peptides^+(PL,SF)^)

**Figure 2:**
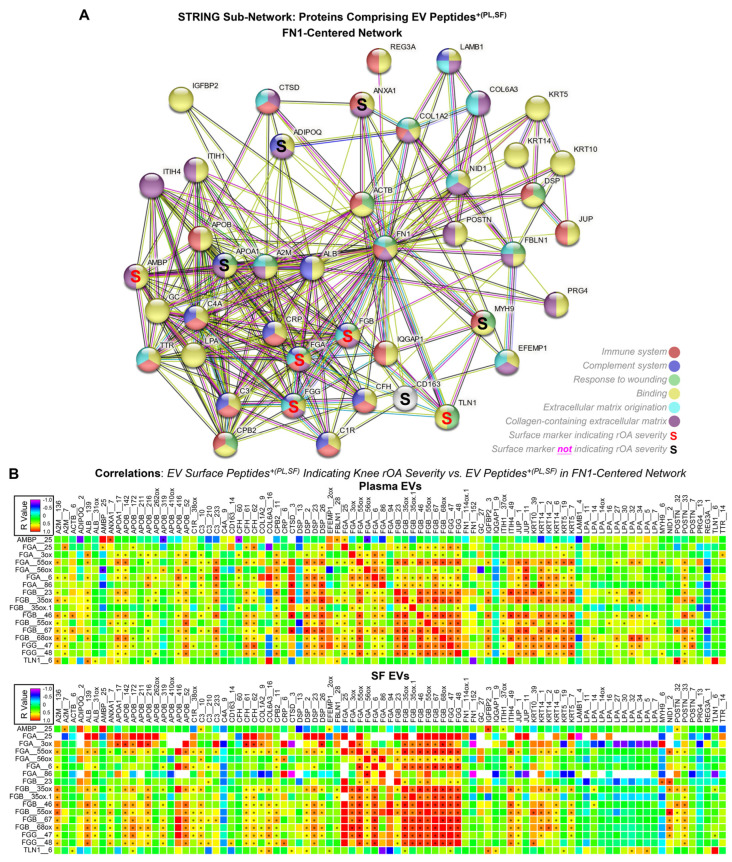
The FN1-centered network. (**A**) The graph displays the 43 proteins in te FN1-centered network comprising EV peptides^+(PL,SF)^ identified in patients with knee OA (n = 16); the processes represented by the colors of each node in the network are detailed in the legend. The edges illustrate protein associations, both functional and physical, all supported by evidence. The reported pathways exhibited an enrichment q < 0.05. (**B**) Spearman correlations were performed to analyze the associations of the EV surface peptides^+(PL,SF)^ indicating knee rOA severity with EV peptides^+(PL,SF)^ in the FN1-contered network in plasma EVs and SF EVs, respectively. Heat maps illustrate the correlation coefficients (r values); * indicated a significant result (*p* < 0.05).

Among the 692 pathogenic SF EV peptides we recently identified as being positively associated with disease severity in OA, 57.4% originated from the immune system, including 19.2% from fibronectin (FN1) and 12.7% from FGA, FGB, and FGG [33]. Interestingly, based on the STRING network analysis [41], FN1 directly interacts with 43 proteins corresponding to 51.2% of the EV peptides^+(PL,SF)^ identified in the current study, forming an FN1-centered network. Of the proteins in the FN1-centered network, 21 were associated with the top Reactome pathway, “Immune System” (Figure 2A). More importantly, all five newly identified EV surface markers—AMBP, FGA, FGB, FGG, and TLN1—consisting of EV surface peptides ^+(PL,SF)^ indicating knee rOA severity (Table 1), were part of this FN1-centered network (Figure 2A). 

Tabula Sapiens [44,45], a whole-body cell atlas that was constructed using single-cell RNA sequencing (scRNA-seq) data of 24 organs from healthy humans (n = 15), revealed a similar tissue/cell distribution among *FGA*, *FGB*, *FGG*, and *AMBP*, and a ubiquitous expression of *TLN* (Figure S2). Some FN1 expressing cells co-expressed these knee rOA severity indicators, especially *FGA*, *FGB*, and *FGG* (Figure S2). In both plasma EVs and SF EVs, FGA, FGB, and FGG peptides^+(PL,SF)^ showed a significant positive correlation with the majority of peptides^+(PL,SF)^ in the FN1-centered network (Figure 2B). 

### 2.4. FN1, ITGB1 (CD29), and TLN1 Genes Expressed in OA Chondrocytes and Synovial Cells

Based on further analyses of our published scRNA-seq data [46] generated from joint tissue cells from patients with end-stage knee OA, the *FN1* gene was expressed by 100%, 99.8%, and 96.1% of damaged-cartilage-derived chondrocytes, intact-cartilage-derived chondrocytes, and synoviocytes, respectively (Figure S3A,B). Similarly, the *ITGB1* gene encoding the FN1 receptor *CD29* (aliases fibronectin receptor subunit beta and integrin β1 subunit) [47] was expressed by 84.9%, 70.7%, and 87.0% of damaged-cartilage-derived chondrocytes, intact-cartilage-derived chondrocytes, and synoviocytes, respectively (Figure S3A,B). 

Among the genes encoding the five identified surface markers (*w*), the *TLN1* gene was expressed by 45.9%, 36.9%, and 50.6% of damaged-cartilage-derived chondrocytes, intact-cartilage-derived chondrocytes, and synoviocytes, respectively (Figure S3C,D), suggesting that both chondrocytes and synoviocytes from end-stage knee OA joint tissue can produce TLN1^+^ EVs. Interestingly, we rarely detected the expression of *AMBP*, *FGA*, *FGB*, or *FGG* genes in OA joint tissue cells (Figure S3C), suggesting that EVs carrying these surface markers likely migrate from the periphery to the OA joint. 

### 2.5. Baseline Frequencies of FGA^+^, FGB^+^, FGG^+^, TLN1^+^, and AMBP^+^ Plasma EV Subpopulations Predicted Progression of Knee rOA

**Figure 3:**
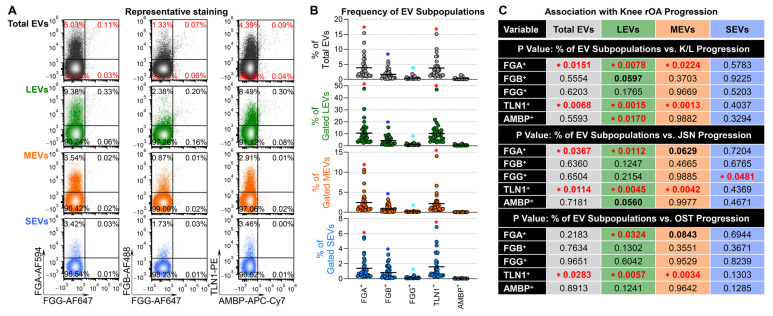
Plasma FGA^+^ and TLN1^+^ EV subpopulations were linked to the progression of knee rOA. EVs from the baseline plasma of patients with knee OA (n = 30) were evaluated for the frequencies of FGA, FGB, FGG, TLN1, and AMBP using flow cytometry. (**A**) The plots show the signals of the tested biomarkers in total plasma EVs and the gated EV subsets (LEVs, MEVs, and SEVs). (**B**) The scatter dot plots illustrate the frequencies of plasma EV subpopulations that carry individual surface markers. We conducted statistical analyses using the Friedman test followed by Benjamini and Hochberg (BH) multiple comparisons, defining statistical significance as a * q < 0.05; red * indicates FGA^+^ and TLN1^+^ subpopulations with significant difference compared to FGB^+^, FGG^+^, and AMBP^+^ subpopulations; dark blue * indicates FGB^+^ subpopulations with significant difference compared to FGG^+^ and AMBP^+^ subpopulations; light blue * indicates FGG^+^ subpopulations with significant difference compared to AMBP^+^ subpopulations. (**C**) Multivariable linear regression modeling, adjusting for baseline demographic and radiographic variables, was performed to identify the associations of the percentages (%) of EV subpopulations carrying the indicated surface markers with the progression of knee rOA. The table displays the *p* values for the associations, with significance defined as * *p* < 0.05 (red font).

Using high-resolution flow cytometry with a surface staining protocol, we detected FGA, FGB, FGG, TLN1, and AMBP with different frequencies on EVs of all sizes (see methods and Figure S1 for size estimation) in the plasma of patients with knee OA (Figure 3A). In all sizes of EVs, FGA^+^ and TLN1^+^ EVs were more abundant than FGB^+^, FGG^+^, and ABMP^+^ EVs, FGB^+^ EVs were more abundant than FGG^+^ and ABMP^+^ EVs, while ABMP^+^ EVs were rare (Figure 3B). Baseline demographic (age, body mass index [BMI], and sex) and radiographic variables (Kellgren Lawrence [K/L] grades, joint space narrowing [JSN], and osteophyte number and size [OST]) were correlated with the frequency of some plasma EV subpopulations carrying the tested surface markers (Figure S4). Therefore, we used multivariable linear regression modeling, adjusting for baseline demographic and radiographic variables, to evaluate the associations between the baseline frequencies of plasma EV subpopulations carrying individual surface markers and the progression of knee rOA from baseline to the 1.1- to 8.6-year follow-up timepoint. Significant associations were observed between higher baseline percentages of several EV markers and OA progression measures, these included: TLN1^+^ subpopulations of all sizes and FGA^+^ LEV subpopulations with all three progression outcomes; FGA^+^ total EV subpopulations with K/L and JSN progression; and FGA^+^ MEVs with K/L progression (Figure 3C).

Regardless of size, the baseline percentages of most individual plasma EV subpopulations carrying FGA, FGB, FGG, TLN1, and AMBP predicted the progression of knee rOA (defined as any unit increase in K/L, JSN, and OST scores) with a range of the areas under the receiver operating characteristic (ROC) curve (AUC) of 0.556–0.711; the exception was AMBP^+^ MEVs (AUC 0.463) (Table 2A). The top 5 individual predictors of knee rOA progression were the baseline percentages of the following: FGA^+^ total EVs; FGA^+^, FGG^+^, and TLN1^+^ LEVs; and AMBP^+^ SEVs (range of AUCs 0.671–0.711, Table 2A). Overall, individual LEV subpopulations yielded higher AUCs than the corresponding individual MEV and SEV subpopulations (Table 2A).

Except for the FGG^+^ LEV and MEV combination, which had a lower AUC than FGG^+^ LEVs alone, the combination of the LEV and MEV individual predictors with the same surface markers was more discriminative of knee rOA progressors from non-progressors than the LEV individual predictors alone (Table 2B). Only AMBP^+^ SEVs contributed to a better AUC in combination with AMBP^+^ LEVs and MEVs, while other SEV subpopulations had either the same or lower AUC (Table 2B). These results further support the identification of LEV subpopulations as potentially pathogenic and strong predictors of knee rOA progression.

## 3. Discussion

SF is an ultrafiltrate of plasma containing EVs and other components from peripheral and joint tissues and cells, and therefore reflects not only joint pathology but also systemic alterations that may mediate OA [20,48,49,50]. In our prior study [33], we found that SF EVs carried a 4.9-fold higher number of rOA-associated peptides than plasma EVs, supporting the idea that SF EVs may serve as more reliable indicators of knee rOA severity. However, the collection of SF biospecimens is generally restricted to individuals with joint effusion and causes potential risks of bleeding and infection due to needle insertion into the joint space. Therefore, our objective in this study was to identify EV surface markers indicating rOA severity in the “minimally invasive” biospecimen, plasma, and to assess whether plasma EVs carrying these surface markers could predict rOA progression. In patients with knee OA, we identified 199 EV peptides^+(PL,SF)^ in plasma EVs that positively correlated with levels in corresponding SF EVs. This suggests that EVs carrying these peptides^+(PL,SF)^ actively migrate or exchange between plasma and SF, reflecting the pathogenic status of OA in the joint. Among these newly identified EV peptides^+(PL,SF)^, seven peptides^+(PL,SF)^ from five surface markers (FGA, FGB, FGG, TLN1, and AMBP) met our selection criteria for potentially pathogenic systemic EV biomarkers, showing associations with OA disease severity and surface localization. Using an independent cohort of knee OA patients and a different method (flow cytometry), we validated the presence of these surface markers on plasma EV subpopulations. Moreover, our new analysis revealed that the baseline frequencies of plasma EV subpopulations, particularly those LEVs carrying FGA, FGG, and TLN1 on their surface, independently predicted the progression of knee rOA. Individual LEV subpopulations carrying these knee rOA-indicating surface markers had overall better discriminant capability to predict knee rOA progression than the corresponding MEV and SEV subpopulations. Our data indicate that these accessible EV markers are promising candidate biomarkers of knee OA severity and progression.

A total of 43 proteins, which corresponded to 51.2% of the EV peptides^+(PL,SF)^, including all 5 knee rOA severity-indicating surface markers, constituted an FN1-centered network, highlighting the crucial pathogenic roles of FN1 in OA. FN1 expressing cells co-expressed these knee rOA severity-indicating surface markers, especially FGA, FGB, and FGG [44,45]. Consistently, in both plasma and SF, EV peptides^+(PL,SF)^ of FGA, FGB, and FGG were positively associated with most EV peptides^+(PL,SF)^ in this FN1-centered network. FN1 and FN1 fragments increase in OA SF and are known to contribute to OA pathogenesis and progression [51,52,53,54]. According to our scRNA-seq data, genes encoding FN1 and its receptor ITGB1 (CD29) were expressed at high levels in the OA joint tissue cells [46]. Beyond joints, the *FN1* gene is ubiquitously expressed by various cells [44,45].

Among the genes encoding the five identified surface markers, only *TLN1* was highly expressed in the chondrocytes and synoviocytes of OA joint tissue [46]. In contrast, gene expression of *FGA*, *FGB*, *FGG*, and *AMBP* in the tested OA joint tissue cells was either rare or below the detection limits; however, according to the Human Protein Atlas [43] and Tabula Sapiens [44,45], they are predominantly expressed by hepatocytes. TLN1 binds to CD29, an integrin β1 subunit [55], that we previously reported to be in higher amounts in SF EVs compared with plasma EVs of patients with knee OA [20], and increases the binding affinity of FN1 and CD29 [56]. Integrins play essential roles mediating cell adhesion to the extracellular matrix; integrin dysfunction, including increased levels of integrins α1β1, α2β1, α3β1, α4β1, α5β1, and α6β1 in OA cartilage, is involved in OA pathogenesis [57]. Unlike the well-documented pathogenic roles of FN1 in OA [51,52,53,54], the roles of TLN1 in OA are still largely unknown. Consistent with our finding of *TLN1* gene expression in end-stage OA joint tissues (chondrocytes and synoviocytes), a previous study reported TLN protein expression on chondrocytes in human OA cartilage whose expression was associated with OA disease severity [58]. TLN is a key component of the mechanosensory system in chondrocytes; blocking TLN1 partially reduced cartilage degeneration and eliminated the influence of mechanical stress [58]. Our observations indicated that increased percentages of TLN1^+^ subpopulations in total plasma EVs and subsets (LEVs and MEVs) at baseline were linked to the progression of knee rOA.

The genes *FGA*, *FGB*, and *FGG* encode the corresponding alpha chain Aα, beta chain Bβ, and gamma chain γ, which assemble into the large fibrinogen glycoprotein (AαBβγ)_2_ hexamer [59]. Hepatocytes are the primary source of fibrinogen [59], which can bind to multiple integrins, including CD29, the FN1 receptor [60]. Fibrinogen in EVs binds to the integrin β expressed on macrophages, which exacerbates the inflammatory effects on these cells [61]. We observed varying frequencies of EVs carrying FGA, FGB, and FGG in OA plasma. However, end-stage OA joint tissue cells rarely expressed *FGA*, *FGB, FGG*, and *AMBP*, suggesting that EVs carrying these surface markers were released from non-joint tissue cells (such as hepatocytes) to plasma, and then migrated from the periphery to the OA joint. Plasma EV peptides^+(PL,SF)^ of FGA, FGB, and FGG, i.e., with correlation of plasma and SF EV concentrations, further supported the migration of these non-OA joint tissue cell-produced EVs from the periphery to OA joints. We identified 88 knee OA severity-indicating peptides corresponding to FGA, FGB, and FGG in the SF EVs of knee OA patients [33], which can affect inflamed joints after being immobilized on a surface of damaged tissue [60]. Our findings align with earlier studies that report abundant deposition of fibrinogen and fibrin and their association with arthritis severity in human joints with OA, rheumatoid arthritis, and experimental arthritis [62,63,64]. Based on these findings, FGA^+^ EVs may be one of the best early OA markers and predictors of OA progression. Indeed, we observed an association of higher baseline percentages of EV subpopulations carrying FGA, FGA, and FGG in plasma with knee rOA progression, prominently FGA^+^ and FGG^+^ LEVs (AUC 0.671 and 0.711, respectively). Extravascular fibrinogen deposition and interaction with neutrophils and macrophages, the major infiltrating immune cells and contributors of SF EVs in OA joints [20,65], lead to local inflammation and tissue damage [60]. The circulating half-life of fibrinogen is only around 4 days [59], and expression or immobilization on the surface of EVs may facilitate fibrinogen stabilization and deposition. 

There were some limitations of this study. We selected polymer-based precipitation with high yield and preservation of EV integrity to separate EVs from small volumes of precious human biofluids [66]. As extensively discussed in our previous publications [4,33], this method may result in co-precipitation of non-EV particles. Nevertheless, our study mainly focused on surface markers, unrelated to well-known non-EV particles, with flow cytometric validation of their presence on the EV surface differentiated by EV size. We also analyzed many EV peptides in a relatively small human cohort. To address these limitations, we used other human cohorts and methodologies to validate the potential tissue and cell expressing genes encoding these surface markers and evaluated their associations with OA disease severity and progression. Future studies of cohorts with a larger sample size would be necessary to validate these results. As a semiquantitative method, the results of flow cytometry are affected by the resolution of flow cytometers, parameter settings (such as threshold, voltage, and compensation), and background determination. However, high-resolution flow cytometry simultaneously measures size distribution, internal complexity, and an array of markers (numbers depending on flow cytometer models), therefore it can provide multidimensional results, especially the co-expression of multiple markers with size parameters to provide high confidence that our reported findings are specific to EVs.

**Figure 4:**
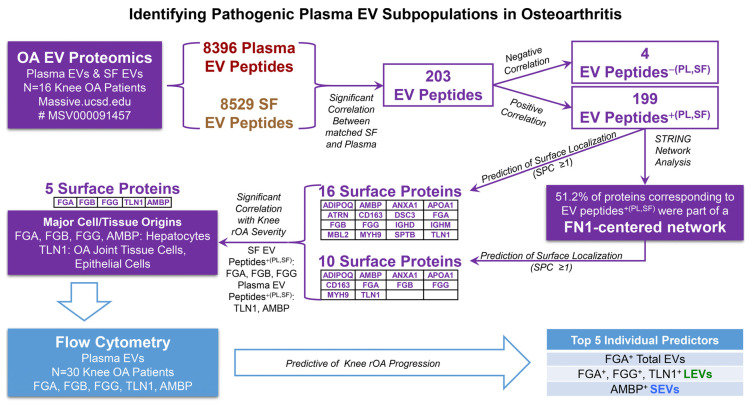
Summary. We selected the potentially pathogenic systemic EV candidates in OA plasma based on the following criteria: (1) a significantly positive association with the corresponding SF EV peptides, termed peptides^+(PL,SF)^; (2) predicted localization to the cell membrane; (3) association with OA disease severity based on our previous report [33]; (4) confirmed surface localization on plasma EVs in another cohort of knee OA patients using flow cytometry; and (5) predictive of knee rOA progression. Using these criteria, we identified potential pathogenic plasma EV subpopulations, especially LEV subpopulations identified by surface markers FGA, FGB, FGG, TLN1, and AMBP, that predict knee rOA progression.

In summary, surface markers of EVs indicate their parent cell origin and therefore can serve as accessible targets for OA therapy, for isolating beneficial EV subpopulations as autologous biological products, or for targeting pathogenic EV subpopulations for elimination. In the present study, we identify potential pathogenic plasma EV subpopulations, especially LEV subpopulations identified by surface markers FGA, FGB, FGG, TLN1, and AMBP, that predict knee rOA progression (Figure 4). Our findings establish a foundation for further development of prognostic biomarkers of knee OA progression based on plasma EV subpopulations, characterized by their specific surface markers, and warrant further study to determine if clearance or prevention of their joint migration might be of therapeutic benefit in OA.

## 4. Materials and Methods

### 4.1. Study Cohorts

Knee rOA severity was evaluated by three types of radiographic scores including K/L, JSN, and OST [65]. Demographic and radiographic scores for the sample sets were as follows: (1) proteomic cohort: our published proteomic data (dataset # MSV000091457 at Massive.ucsd.edu) [33] were derived from matched plasma EVs and SF EVs from patients with knee OA (n = 16; age 69 ± 12 years; BMI 33 ± 9 kg/m^2^, 50% female; index knee [providing SF] K/L 2–4, summed [for both knees] K/L 2–8; index knee JSN 0–4, summed JSN 0–9; index knee OST 2–10, summed OST 2–19). This cohort was used in our previous studies assessing 18 surface markers and 4 proinflammatory cytokines [20], and knee rOA severity associated plasma EVs and SF EVs [33]. (2) Flow cytometry cohort: plasma specimens were collected from the participants with knee OA in the completed Genetics of Generalized Osteoarthritis (GOGO) study [67] (n = 30; age 69 ± 8 years; BMI 31 ± 7 kg/m^2^; 53% female; knee OA scores summed K/L 1–6; summed JSN 0–4; summed OST 0–5). Knee rOA progression was defined as an increase in ≥1 unit in K/L, JSN, or OST grade in at least one knee during the follow-up period (mean 3.8 years, range 1.1–8.6 years). The summed rOA progression scores for both knees were as follows: K/L 0–6, JSN 0–11, and OST 0–6. This cohort was used in our previous study assessing immune cell- and proinflammatory cytokine-related plasma EV subpopulations in predicting knee OA progression [28]. All biospecimens were collected with informed consent and approved by the Institutional Review Board (IRB) at Duke University.

### 4.2. EV Isolation

As we previously reported [6,20,33], freshly collected whole blood and SF biospecimens were centrifuged (3000 rpm, 15 min, 4 °C) to remove cells, platelets, and debris. The biofluids were subsequently aliquoted and frozen at −80 °C. Before EV isolation, the thawed biofluids were centrifuged (2000× *g*, 10 min, 4 °C) to eliminate unwanted material and debris. Then, EVs were separated from the cleaned plasma and SF (digested in hyaluronidase [Sigma-Aldrich, St. Louis, MI, USA], 10 unit/mL, 1 h, 37 °C) using polymer-based precipitation (ExoQuick, System Biosciences, Palo Alto, CA, USA) according to the manufacturer’s instructions [6,20,33]. 

### 4.3. Analyses of OA EV Proteomic Data

Our published OA plasma and SF EV proteomic data [33] were generated by the Duke Proteomics and Metabolomics Core Facility, using a nanoAcquity UPLC system (Waters Corp., Milford, MA, USA) coupled to an Orbitrap Fusion Lumos high-resolution accurate mass tandem mass spectrometer equipped with a FAIMS Pro system (Thermo Fisher Scientific, Waltham, MA, USA). We identified EV peptides^+(PL,SF)^ and predicted their surface localization based on their SPC score generated using SurfaceGenie (https://www.cellsurfer.net/surfacegenie, accessed on 16 July 2021) [42] as we previously reported [33]; the membrane localization was supported by STRING network analysis [41]. Tabula Sapiens [44,45] facilitated the identification of cells expressing the genes that encode the identified surface proteins. STRING network analysis [41] was conducted to examine the interactions and functional enrichment pathways of the identified EV biomarkers. The network was generated using the query proteins only, with a minimum required interaction score of 0.4 indicating medium confidence. The active interaction sources include co-expression, co-occurrence, databases, experiments, gene fusion, neighborhood, and text mining. The reported pathways exhibited an enrichment q < 0.05.

### 4.4. Gene Expression of Biomarkers of Interest in OA Joint Tissue

Our published scRNA-seq data (ID: NCBI GEO GSE152805) [46] was generated from three types of joint tissue cells from patients with end-stage knee OA, including damaged-cartilage-derived chondrocytes, intact-cartilage-derived chondrocytes, and synoviocytes from synovium. We analyzed the genes encoding *FN1* and its binding receptor, along with our identified surface markers, to determine the number and percentage of cells expressing each individual gene. 

### 4.5. High-Resolution Multicolor Flow Cytometry

Following our published research [4,6,20], EV pellets separated from 20 µL plasma specimens were resuspended in double filtered PBS (df-PBS) that was filtered twice through 100 nm filters (EMD Millipore, Temecula, CA, USA) and stained with fluorescence-conjugated-antibodies against human FGA, FGB, FGG, TLN1 (Bio-techne, Minneapolis, MN, USA), and AMBP (LSBio, Newark, CA, USA). Df-PBS and unstained EVs were used as negative controls. To set compensation and define fluorescence background and positive signals, we used negative controls along with EVs and UltraComp™ eBeads plus (ThermoFisher Scientific) stained with each antibody. The frequencies of the tested biomarkers, as defined by the percentages of EVs carrying each biomarker, were measured using a MA900 flow cytometer with 4-way sorting function (Sony Biotechnology, San Jose, CA, USA) and analyzed using FCS Express 5 Flow Research Edition (De Novo Software, Pasadena, CA, USA). According to our reported serial dilutions for determining the optimal dilution factors to avoid swarm detection [4], the final volume of each specimen for flow cytometric analysis was adjusted to 300 µL using df-PBS, resulting in a final dilution factor 1:15 for the original 20 µL plasma used for EV separation. The flow cytometer was configured to acquire samples at around 1 µL/second with a threshold BSC (back scatter, also known as side scatter [SSC]) 0.02% to exclude small debris particles but keep the capacity to detect small EVs. This threshold setting ensured that the background noise, defined by acquisition events of df-PBS, remained below 10 events per second. Notably, as of now, the scatter resolution of all flow cytometers in the markets (nanoscaled or conventional) is not good enough to fully separate EVs, especially small EVs, from the background noise using only scatter (forward scatter [FSC] and SSC/BSC). Size (FSC) and granularity (SSC) were useful to display the relative size distribution of EVs for gating purposes. However, it is necessary to use fluorescence-conjugated antibodies to catch EVs with specific markers, to separate them from the background noise. We estimated EV sizes using non-fluorescent size reference beads with mean diameters of 100 nm (3000 Series Nanosphere™ Size Standards), 1000 nm (8000 Series Silica Particle Size Standards), and 6000 nm (Duke Standards™ 2000 Series Uniform Polymer Particles, ThermoFisher Scientific), allowing us to differentiate LEVs (~1000–6000 nm), MEVs (~100–1000 nm), and SEVs (~100 nm or smaller) based on the Forward Scatter Height (FSC-H) distribution from the flow cytometry profiles (Figure S1). As extensively discussed in our previous studies [4,6,33], the estimation of biological vesicle sizes using artificial size reference beads can be influenced by numerous factors; therefore, our reported EV sizes are approximations rather than exact measurements. Despite this, the size heterogeneity and relative distribution of EVs identified through flow cytometry were verified by dynamic light scattering [6] and transmission electron microscopy [4]. 

### 4.6. Statistical Analyses

The statistical analyses conducted in this study included: (1) Pearson analyses for assessing the univariate associations of each peptide’s expression in matched plasma EVs and SF EVs; (2) Spearman analyses for assessing associations of the identified EV surface peptides^+(PL,SF)^ with other peptides^+(PL,SF)^; (3) Friedman test followed by BH multiple comparisons for comparing the frequencies of plasma EV subpopulations that carry individual tested surface markers; (4) multivariable linear regression modeling, adjusting for baseline demographic and radiographic variables, to identify associations of the tested plasma EV subpopulations with knee rOA progression [68]; and (5) multivariable logistic regression and ROC curve analyses [68] to assess the discriminant capacity of the tested baseline EV biomarkers for predicting knee rOA progression. Validation of the predictive models was performed using a Bootstrap method, repeating the analysis 2500 times with different resampling weights. AUCs and 95% Bootstrap Bias-Corrected Confidence Limits (upper, lower) are reported. The AUCs varied between 0.5 and 1, suggesting discrimination between patients with and without knee rOA progression, where higher AUC values correspond to improved discrimination [69,70]. Statistical significance was defined as *p* < 0.05 or q < 0.05, where applicable.

## Figures and Tables

**Table 1:** Surface markers comprising 29 EV peptides^+(PL,SF)^. The table displays 29 EV peptides^+(PL,SF)^ corresponding to 16 proteins with predicted surface localization that were defined as SPC ≥ 1 (more details in Supplementary Table S1). Positive associations of the indicted EV peptides with knee rOA severity scores (JSN or OST) were recently reported [33].

Peptide Name	Gene Name	Protein Name	Peptide Sequence	SPC	Accession Number	Protein Position(s)	Plasma vs. SF Spearman R Value	Indicating Knee rOA Severity
ADIPOQ__2	*ADIPOQ*	Adiponectin	GDIGETGVPGAEGPR	1	Q15848	Q15848 (78–92)	0.9511	
AMBP__25	*AMBP*	Protein AMBP	VVAQGVGIPEDSIFTMADR	1	P02760	P02760 (167–185)	0.8152	**Yes (in plasma, JSN)**
ANXA1__7	*ANXA1*	Annexin A1	GGPGSAVSPYPTFNPSSDVAALHK	1	P04083	P04083 (30–53)	0.9701	
APOA1__17	*APOA1*	Apolipoprotein A-I	EQLGPVTQEFWDNLEK	1	P02647	P02647 (86–101)	0.7908	
ATRN__3	*ATRN*	Attractin	CTWLIEGQPNR	4	O75882	O75882 (158–168)	0.8627	
CD163__14	*CD163*	Scavenger receptor cysteine-rich type 1 protein M130	QLGCGSALK	4	Q86VB7	Q86VB7 (413–421)	0.9858	
DSC3	*DSC3*	Desmocollin-3	IPIEDKDLINTANWR	3	Q14574	Q14574 (376–390)	0.9178	
FGA__25	*FGA*	Fibrinogen alpha chain	GDFSSANNR	1	P02671	P02671 (115–123)	0.9889	
FGA__3ox	*FGA*	Fibrinogen alpha chain	AQLVDMKR	1	P02671	P02671 (161–168)	0.8382	
FGA__55ox	*FGA*	Fibrinogen alpha chain	MKGLIDEVNQDFTNR	1	P02671	P02671 (70–84)	0.5799	**Yes (in SF, OST)**
FGA__56ox	*FGA*	Fibrinogen alpha chain	MKGLIDEVNQDFTNRINK	1	P02671	P02671 (70–87)	0.8833	
FGA__6	*FGA*	Fibrinogen alpha chain	DNTYNRVSEDLR	1	P02671	P02671 (124–135)	0.5649	
FGA__86	*FGA*	Fibrinogen alpha chain	SYKMADEAGSEADHEGTHSTKR	1	P02671	P02671 (600–621)	0.9191	
FGB__23	*FGB*	Fibrinogen beta chain	HQLYIDETVNSNIPTNLR	1	P02675	P02675 (179–196)	0.6397	**Yes (in SF, OST)**
FGB__35ox	*FGB*	Fibrinogen beta chain	MGPTELLIEMEDWKGDKVK	1	P02675	P02675 (335–353)	0.5589	**Yes (in SF, OST)**
FGB__35ox.1	*FGB*	Fibrinogen beta chain	MGPTELLIEMEDWKGDKVK	1	P02675	P02675 (335–353)	0.8016	
FGB__46	*FGB*	Fibrinogen beta chain	QGFGNVATNTDGKNYCGLPGEYWLGNDKISQLTR	1	P02675	P02675 (301–334)	0.6003	**Yes (in SF, OST)**
FGB__55ox	*FGB*	Fibrinogen beta chain	TMTIHNGMFFSTYDRDNDGWLTSDPRK	1	P02675	P02675 (396–422)	0.6253	
FGB__67	*FGB*	Fibrinogen beta chain	YQISVNKYR	1	P02675	P02675 (368–376)	0.5434	
FGB__68ox	*FGB*	Fibrinogen beta chain	YYWGGQYTWDMAK	1	P02675	P02675 (446–458)	0.5589	
FGG__47	*FGG*	Fibrinogen gamma chain	RLDGSVDFKK	1	P02679	P02679 (223–232)	0.5073	**Yes (in SF, OST)**
FGG__48	*FGG*	Fibrinogen gamma chain	TRWYSMK	1	P02679	P02679 (400–406)	0.6007	
IGHD__1	*IGHD*	Immunoglobulin heavy constant delta	STTFWAWSVLR	1	P01880	P01880 (331–341)	0.9619	
IGHM__9	*IGHM*	Immunoglobulin heavy constant mu	ESDWLGQSMFTCR	1	P01871	P01871 (186–198)	0.9940	
IGHM__9ox	*IGHM*	Immunoglobulin heavy constant mu	ESDWLGQSMFTCR	1	P01871	P01871 (186–198)	0.9938	
MBL2__1	*MBL2*	Mannose-binding protein C	ALQTEMAR	1	P11226	P11226 (114–121)	0.9765	
MYH9__6	*MYH9*	Myosin-9	QTLENERGELANEVK	1	P35579	P35579 (1220–1234)	0.7950	
SPTB__12	*SPTB*	Spectrin beta chain, erythrocytic	QLMDEKPQFTALVSQK	1	P11277	P11277 (1338–1353)	0.9935	
TLN1__6	*TLN1*	Talin-1	EVANSTANLVK	1	Q9Y490	Q9Y4G6 (1533–1543); Q9Y490 (1531–1541)	0.6008	**Yes (in plasma, OST)**

**Table 2:** Predictors of knee rOA progression. The plasma EVs from participants with knee OA (n = 30) at baseline were profiled for the identified surface markers by high-resolution flow cytometry. Multivariable logistic regression and ROC curve analyses were used to evaluate the discriminant ability of the tested baseline plasma EV subpopulations for any unit knee rOA progression (defined as any unit increase in K/L, JSN, or OST scores from baseline to follow-up). (**A**,**B**) The table displays AUC and 95% Bootstrap Bias-Corrected Confidence Limits (upper, lower) for the individual predictors (**A**) or combination of individual predictors with same surface markers (**B**). The font in bold in indicates the top 5 AUCs among the individual predictors of knee rOA progression (**A**) or the higher AUCs compared to the corresponding individual predictors (**B**).

**(A) Individual Predictors**				
**Variables**	**% of Total EVs**	**% of Gated LEVs**	**% of Gated MEVs**	**% of Gated SEVs**
**FGA^+^ Subpopulation**	**0.671** (0.435, 0.868)	**0.671** (0.465, 0.852)	0.620 (0.416, 0.814)	0.556 (0.349, 0.74)
**FGB^+^ Subpopulation**	0.644 (0.442, 0.833)	0.655 (0.479, 0.845)	0.590 (0.435, 0.868)	0.563 (0.403, 0.731)
**FGG^+^ Subpopulation**	0.627 (0.361, 0.835)	**0.711** (0.457, 0.889)	0.565 (0.377, 0.745)	0.639 (0.46, 0.845)
**TLN1^+^ Subpopulation**	0.653 (0.453, 0.840)	**0.678** (0.479, 0.857)	0.606 (0.421, 0.805)	0.604 (0.436, 0.794)
**AMBP^+^ Subpopulation**	0.630 (0.347, 0.841)	0.655 (0.460, 0.847)	0.463 (0.220, 0.585)	**0.697** (0.462, 0.857)
**(B) Combination of Individual Predictors with Same Surface Markers**				
**Variables**	**% of Gated LEVs** **% of Gated MEVs**		**% of Gated LEVs** **% of Gated MEVs** **% of Gated SEVs**	
**FGA^+^ Subpopulations**	**0.694** (0.468, 0.852)		0.690 (0.444, 0.818)	
**FGB^+^ Subpopulations**	**0.731** (0.523, 0.899)		0.731 (0.485, 0.889)	
**FGG^+^ Subpopulations**	0.685 (0.431, 0.824)		0.671 (0.398, 0.768)	
**TLN1^+^ Subpopulations**	**0.704** (0.423, 0.851)		0.704 (0.427, 0.820)	
**AMBP^+^ Subpopulations**	**0.711** (0.458, 0.876)		**0.808** (0.603, 0.929)	

## Data Availability

All data used to evaluate the conclusions in the paper are present in the paper and/or the Supplementary Materials. All proteomic data for this study are available at massive.ucsd.edu (#MSV000091457).

## Supplementary Materials

The supporting information can be downloaded at: https://www.mdpi.com/article/10.3390/ijms252312504/s1.
