# Supplementary material for: Immune System-Related Plasma Pathogenic Extracellular Vesicle Subpopulations Predict Osteoarthritis Progression
Source: Int J Mol Sci. 2024 Nov 21;25(23):12504. doi: 10.3390/ijms252312504 (PMC11641473; doi:10.3390/ijms252312504)
Supplement: Supplementary file 1 [file ijms-25-12504-s001.zip › Supplementary Figures-11192024.pdf]

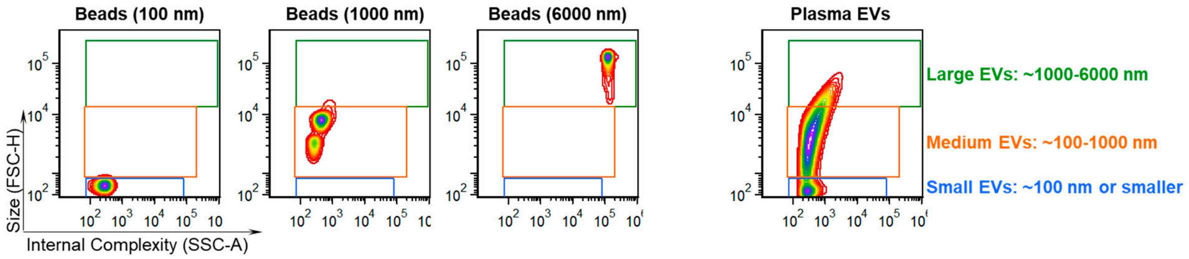

Figure S1. EV size estimation with non-fluorescent reference beads. Non-fluorescent reference beads of mean diameters 100, 1000 and 6000 nm were used for size estimation; the relative size distribution of plasma EVs was defined as follows: large EVs (LEVs), ~1000-6000 nm; medium-sized EVs (MEVs), ~100-1000 nm; and small EVs (SEVs), ~100 nm or smaller. The representative plots present size (FSC-H: Forward Scatter-Height), and internal complexity/granularity (SSC-A: Side Scatter-Area) for size reference beads and OA plasma EVs, and the gating strategy for LEVs, MEVs and SEVs.

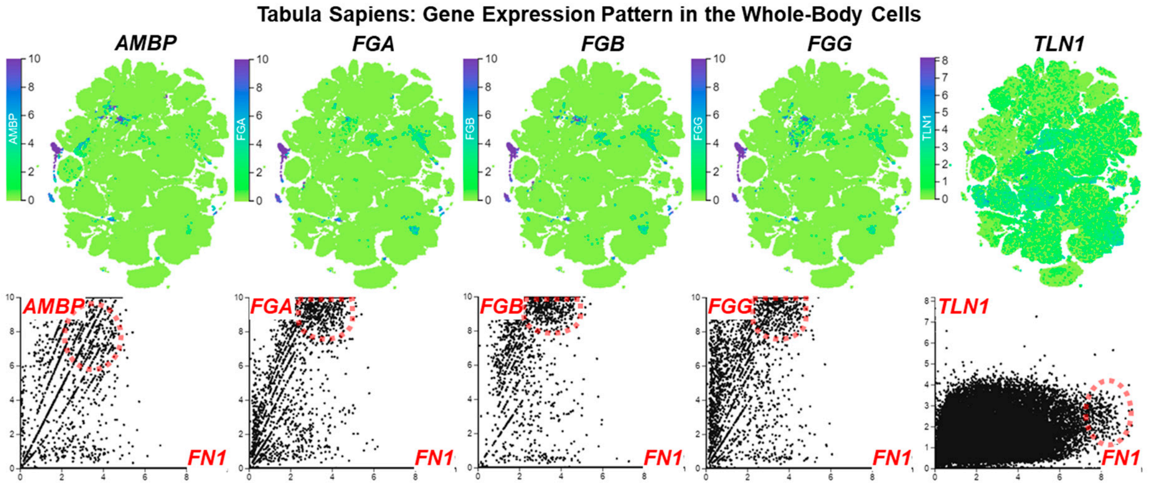

Figure S2. The gene expression pattern of *AMBP*, *FGA*, *FGB*, *FGG*, and *TLN1* in the whole-body cells. Tabula Sapiens were utilized to identify and display the expression pattern of the indicated surface markers using UMAP and their co-expression with *FN1* in the whole-body cells.

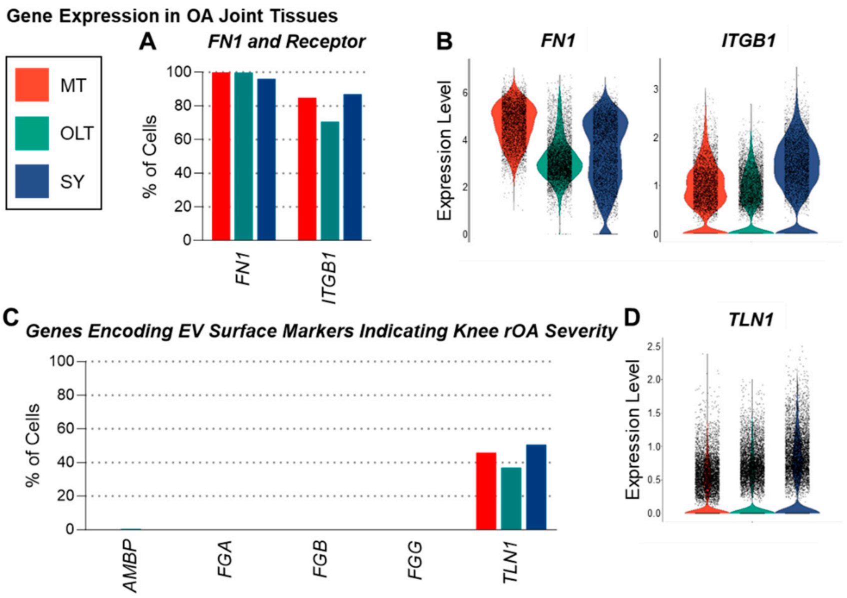

Figure S3. ***FN1, ITGB1,* and *TLN1* genes were highly expressed by chondrocytes and synoviocytes from knee OA joint tissue cells.** ScRNA-seq gene expression data were generated from joint tissues of participants (n=3) with end-stage knee OA undergoing joint replacement surgery, including 11,579 chondrocytes from damaged sites of the medial tibial cartilage (MT), 14,613 chondrocytes from intact sites of the outer lateral tibial cartilage (OLT), and 10,640 synoviocytes from the matched synovium (SY). The data were evaluated for number of cells expressing the indicated genes. **A, C,** the bar graph displays the % of cells expressing the indicated genes in cells of MT, OLT and SY. **B, D,** the violin (density) plots display the expression level of the indicated gene in the three types of OA joint tissue cells.

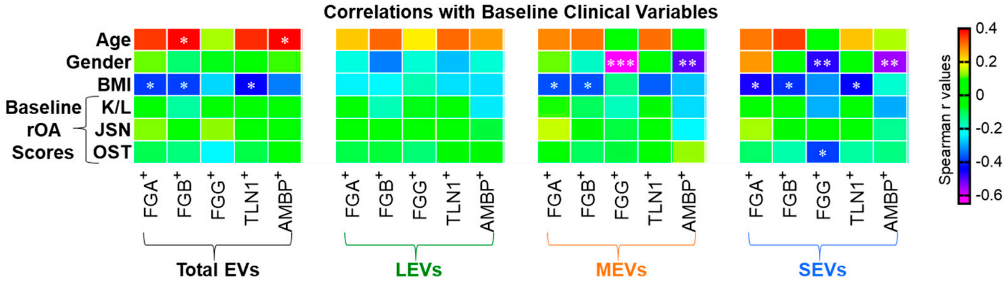

Figure S4. Baseline clinical variables affected the frequency of plasma EV subpopulations. EVs were separated from plasma samples of patients with knee OA (n=34) at baseline and profiled for the identified surface markers by high-resolution flow cytometry. Spearman analysis was performed to evaluate the correlations of the baseline percentages of the EV subpopulations carrying the indicated surface markers with clinical variables (age, gender, BMI, summed knee OA K/L, JSN and OST scores). Heat maps depict Spearman correlation coefficient r values; significant results were defined by * $p<0.05$; ** $p<0.01$, *** $p<0.001$.
